# Influencing factors of dexmedetomidine on postoperative myocardial protection in patients with Video-Assisted Thoracoscopic Surgery lobectomy: A retrospective cohort study

**DOI:** 10.12669/pjms.39.3.7594

**Published:** 2023

**Authors:** Hua Li, Ji Liu, Chun Li, Hong Shi

**Affiliations:** 1 Hua Li, Department of Anesthesiology, Shanghai Pulmonary Hospital, Tongji University, School of Medicine, Shanghai 200433, P.R. China; 2 Ji Liu, Department of Anesthesiology, Shanghai Pulmonary Hospital, Tongji University, School of Medicine, Shanghai 200433, P.R. China; 3 Chun Li, Department of Anesthesiology, Shanghai Pulmonary Hospital, Tongji University, School of Medicine, Shanghai 200433, P.R. China; 4 Hong Shi, Department of Anesthesiology, Shanghai Pulmonary Hospital, Tongji University, School of Medicine, Shanghai 200433, P.R. China

**Keywords:** Dexmedetomidine, Thoracoscopic Surgery lobectomy, Postoperative analgesia effect, Length of hospital stay

## Abstract

**Objective:**

To investigate factors affecting the cardioprotective proprieties of dexmedetomidine in patients after pulmonary lobectomy.

**Methods:**

The data of 504 patients who received dexmedetomidine combined with general anesthesia and video-assisted thoracoscopic surgery (VATS) lobectomy in Shanghai Lung Hospital from April 2018 to April 2019 were analyzed retrospectively. Patients were divided into normal troponin group (LTG) and high troponin group (HTG) according to whether the postoperative troponin level was greater than 13. Ratio of systolic blood pressure greater than 180, heart rate greater than 110, the dose of dopamine and other drugs, the ratio of neutrophils to lymphocytes, postoperative visual analog scale (VAS) pain score and hospital stay were compared between the two groups.

**Results:**

Preoperative systolic blood pressure, intraoperative maximum systolic blood pressure, intraoperative maximum heart rate, intraoperative minimum heart rate and N-terminal pro hormone brain natriuretic peptide (NT-proBNP) correlated with troponin values. The proportion of patients with systolic blood pressure greater than 180 in the HTG was higher than that in the LTG (p=0.0068), and the proportion of patients with heart rate greater than 110 was significantly higher in the HTG compared to the LTG (p=0.044). The ratio of neutrophils/lymphocytes in the LTG was lower than that in the HTG (P<0.001). At 24 and 48 hours after operation, the VAS score in the LTG was lower than that in the HTG. Patients with high troponin had longer hospital stay.

**Conclusions:**

Intraoperative systolic blood pressure, maximum heart rate, and postoperative neutrophil/lymphocyte ratio are important factors that affect the myocardial protection properties of dexmedetomidine and may affect the postoperative analgesia effect and the length of hospital stay.

## INTRODUCTION

Dexmedetomidine is an α2 adrenergic receptor agonist with various pharmacological effects including analgesia and anxiolytic.^1^ Studies have also found that dexmedetomidine has cardioprotective effect.^2^ Pharmacological animal studies have confirmed that dexmedetomidine exerts a cardioprotective effect by activating α2 adrenergic receptors, regardless of the timing of the treatment with the agent.^3^,^4^ In recent year, studies have suggested that the cardioprotective mechanism of dexmedetomidine is related to the activation of related signal transduction pathways. Ibacache M et al^5^ pointed out that the activation of extracellular regulated protein kinase 1/2, protein kinase B and endothelial nitric oxide synthase (eNOS) could be induced in rat hearts pretreated with dexmedetomidine.

This led to improved myocardial function and reduced cardiac ischemia-reperfusion injury. Studies have showed that dexmedetomidine protects the heart by inhibiting the expression of high mobility group box one and activating the cholinergic anti-inflammatory pathway.^6^ The results of the bioinformatic analysis suggested that the p53 pathway also plays an important role in this process, but further research is needed to further explore the molecular mechanism of dexmedetomidine’s cardioprotective effect. Riquelme et al^7^and whether this action occurs directly or indirectly on cardiomyocytes, still remain unclear. The endothelial nitric oxide synthase (eNOS studied the effect of dexmedetomidine on the eNOS/NO pathway in ischemia-reperfusion injury rat model. and showed that dexmedetomidine promotes the production and activation of eNOS in endothelial cells, thus reducing myocardial infarction size and improving left ventricular function.^8^

In patients undergoing surgery, hemodynamic fluctuations caused by factors such as surgical stimulation and extracorporeal circulation can lead to adverse events such as aggravation of cardiac insufficiency, arrhythmia, and myocardial ischemia.^9^ It is not known whether these changes during the operation with general anesthesia may affect the cardioprotective effect of dexmedetomidine.^10^ In our study, we retrospectively analyzed the patients undergoing thoracoscopic lobectomy with dexmedetomidine combined with general anesthesia, and observed and analyzed the factors affecting the cardioprotection of dexmedetomidine.

## METHODS

Data of 825 patients who underwent thoracoscopic lobectomy in Shanghai Pulmonary Hospital from April 2018 to April 2019 were retrospectively analyzed. The data were obtained from the electronic medical record system of Shanghai Pulmonary Hospital. Of 825 patients, 535 underwent VATS lobectomy and were considered for the inclusion in the study.

### Exclusion Criteria:

Thirty-one patients were excluded based on the following exclusion criteria:

1. Any history of heart disease, including coronary heart disease, myocardial infarction, arrhythmia, cardiomyopathy, etc.
2. Hypertension that fails to reach the target after drug treatment or untreated.

For the remaining 504 patients, clinical indicators related to the troponin value were analyzed by regression analysis. Patients were then divided into normal troponin group (LTG) and increased troponin group (HTG) according to whether the postoperative troponin level was greater than 13 (Fig.I).

**Fig1:**
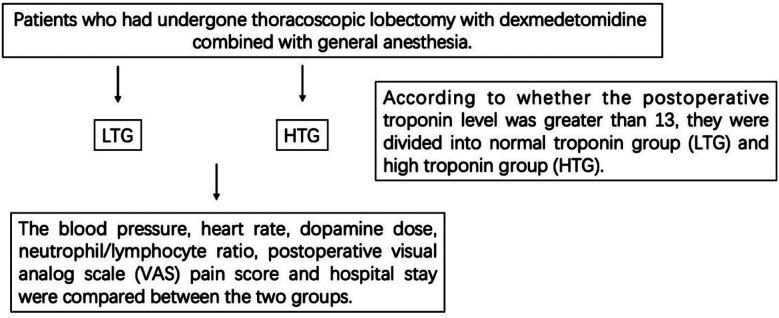
Flowchart of trial procedures.

### Ethical approval:

The study was approved by the ethics committee of the hospital (No. K18-141, Date: 2018-06-11).

The anesthesia regimen of all patients was dexmedetomidine combined with general anesthesia, and no pre-anesthetic medication was used before anesthesia. Patients fasted for 12 hours (water and food) before the operation. Venous access was established in the operating room, and vital signs were monitored. Each patient received a central venous catheter. Then, 1μg/kg of dexmedetomidine was injected intravenously within 10 minutes before induction of anesthesia, and maintained at a rate of 0.2 μg/ (kg·h). After induction of anesthesia, mechanical ventilation was done with endotracheal intubation: tidal volume (VT) 8-10 mL/kg, respiratory ratio 1:2, RR: 12-14 times/minute, and oxygen flow 2 L/min, with continuous injection of 3-6 mg/ (kg·h) propofol, 0. Anesthesia was maintained with 05-0.2 μg/ (kg·min) remifentanil and 1-2 μg/ (kg·min) cisatracurium. The depth of anesthesia is monitored by an anesthesia depth monitor. During the operation, blood oxygen saturation [Sp(O2)] was maintained at about 98%, PETCO2 at 35-45 mmHg (1 mmHg = 0.133 kPa), and Fi (O2) at 0. 8 to 1. 0. Propofol, cisatracurium, and remifentanil were discontinued 10 minutes before skin suture. When the patient resumed spontaneous breathing, consciously opened his eyes, and was conscious, the tracheal intubation was removed. Sp(O2) was maintained at > 95%, an analgesic pump (2 μg/kg sufentanil citrate injection + 100 mL normal saline) was used. Repeated doses of 0.5-2.5 mg neostigmine were injected intramuscularly or subcutaneously at appropriate intervals, with a daily dose range of 5-20 mg and analgesia for two days. Patients self-administered non-steroidal anti-inflammatory drugs (Celebrex) for two days. The following clinical indicators were compared between groups:

- The proportion of patients with intraoperative systolic blood pressure greater than 180 and heart rate greater than 110;
- Intravenous injection of 1-2 μg·kg^-1^ each time during anesthesia can increase heart rate and blood pressure. So we compared the amount of dopamine and other drugs used in anesthesia between the two groups;
- The ratio of neutrophils to lymphocytes in blood samples of patients on the second day after surgery;
- The VAS score of postoperative pain,
- Total length of hospital stay including surgery duration.

### Statistical Analysis:

SPSS 20.0 software was used to analyze the data. Normal distribution measurement data were expressed as *χ̅*±*S*. The comparison between the groups was done by t-test. Within-group comparisons were made by the paired t-test; count data were expressed by case (%). Comparisons between groups were performed using the *χ^2^* test. The correlation between the indicators was analyzed by multiple linear regression. *P* < 0. 05 indicated statistical significance.

## RESULT

Medical records of 634 patients were reviewed, including 534 cases of VATS lobectomy and 100 cases of non-VATS lobectomy. Of 534 VATS-lobectomy cases, 503 were included in this study, and 31 cases were excluded due to preoperative structural heart disease, history of myocardial infarction, history of coronary heart disease, etc. There were 438 cases in the LTG and 65 cases in the increased troponin group. There was no statistical difference in the general demographics such as age, sex ratio, height, and age between the two groups (Table-I).

**Table-I:** General Information of Patients.

	HTG (n=65)	LTG (n=438)	P value
Age (year,)	58.18±10.38	59.84±11.06	>0.05
Gender (Male,%)	72.8%	70.9%	>0.05
Height	167.14±7.90	168.72±7.37	>0.05
Weight	68.04±10.98	69.19±11.02	>0.05

There was no statistical difference in the general demographics such as age, sex ratio, height, and age between the two groups.

We used multiple linear regression analysis to assess the correlation between troponin levels and other observed indicators. The analysis results showed that preoperative systolic blood pressure, intraoperative maximum systolic blood pressure, intraoperative maximum heart rate, intraoperative minimum heart rate, and NT-proBNP correlated with troponin values. The results are summarized in (Table-II).

**Table-II:** Correlation of Troponin with Other Observations.

	Unstandardized Coefficients Beta	Standardized Coefficients Beta	t	P	VIF
Preoperative HR	-0.038	-0.085	-1.647	0.1	1.206
Preoperative SYS	-0.036	-0.128	-2.05	0.041	1.768
Preoperative DIA	0.095	0.225	4.12	<0.001	1.361
Intraoperative maximum HR	0.038	0.099	1.902	0.058	1.233
Intraoperative minimum HR	0.019	0.042	0.792	0.429	1.26
Intraoperative maximum SYS	0.069	0.236	3.534	<0.001	2.039
Intraoperative maximum DIA	0	0.001	0.009	0.993	1.736
Intraoperative minimum SYS	0.017	0.043	0.713	0.476	1.69
Intraoperative minimum DIA	-0.077	-0.135	-2.114	0.035	1.856
NT-proBNP	0.003	0.153	3.177	0.002	1.062
Myoglobin	0.003	0.079	1.551	0.122	1.175
Creatine kinase isoenzyme	-0.027	-0.027	-0.528	0.598	1.204

The results of correlation analysis showed that: preoperative systolic blood pressure, intraoperative maximum systolic blood pressure, intraoperative maximum heart rate, intraoperative minimum heart rate, and NT-proBNP correlated with troponin values. *: p < 0.05.

**Fig.2:**
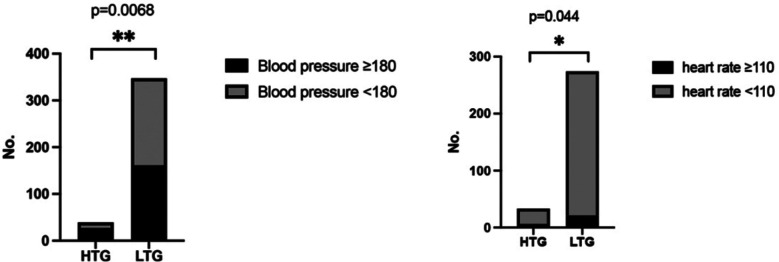
The proportion of intraoperative systolic blood pressure greater than 180 and heart rate greater than 110 The percentage of patients with intraoperative systolic blood pressure greater than 180 and the proportion of patients with heart rate greater than 110 in the HTG was higher than that in the LTG. *: p < 0.05.

The percentage of patients with intraoperative systolic blood pressure greater than 180 in the HTG was higher than that in the LTG (p=0.0068), and the proportion of patients with heart rate greater than 110 was significantly higher than that in the LTG (p=0.044). The results are shown in Fig.2.

We compared the intraoperative doses of dopamine, atropine, esmolol, norepinephrine, and nicardipine between the two groups, and the results showed no significant inter-group difference (Table-III). The ratio of neutrophils/lymphocytes in the LTG was lower than that in the high troponin group, P<0.001.

**Table-III:** Comparison of intraoperative drug dosage and neutrophil/lymphocyte ratio.

	HTG (n=65)	LTG (n=438)	P value
Neutrophil/Lymphocyte ratio	6.43±2.08	3.47±1.22	<0.001
Dopamine	4.60±1.51	3.68±1.80	0.507
Atropine	0.026±0.10	0.12±0.60	0.179
Esmolol	15.86±13.79	16.84±14.55	0.720
Norepinephrine	2.00±1.41	1.49±1.17	0.937
Nicardipine	2.11±1.65	1.67±1.80	0.381

The results of correlation analysis showed that: preoperative systolic blood pressure, intraoperative maximum systolic blood pressure, intraoperative maximum heart rate, intraoperative minimum heart rate, and NT-proBNP correlated with troponin values. *: p < 0.05.

The postoperative analgesic effects in both groups were compared using the VAS score, as shown in Table-IV. The VAS scores in the LTG at 24 and 48 hours after operation were lower than those in the high troponin group. However, there was no difference in the number of analgesic pump compressions between the two groups. Patients in the HTG spent more days in hospital.

**Table-IV:** Comparison of analgesic effects and length of hospital stay.

	HTG (n=65)	LTG (n=438)	P value
Analgesic pump compressions	5.18±1.38	2.61±1.57	0.183
VAS 24 hours after surgery	6.46±5.36	3.17±1.60	0.008
VAS 48 hours after surgery	6.14±5.30	1.85±1.52	0.007
The number of days in hospital	9.18±3.31	5.72±2.01	<0.001

The VAS scores in the LTG at 24 and 48 hours after operation were lower than those in the high troponin group. *: p < 0.05.

## DISCUSSION

The results of this study showed that intraoperative systolic blood pressure, maximum heart rate and postoperative neutrophil/lymph ratio were important factors affecting the myocardial protection proprieties of dexmedetomidine and may also affect the postoperative analgesic effect and the length of hospital stay. Dexmedetomidine is a more selective α2 receptor agonist than clonidine, and can reduce the release of excitatory transmitters from the central sympathetic nerve, and exert anxiolytic, sedative and analgesic effects.^11^ α2 receptor agonists have significant advantages in stabilizing the circulatory dynamics due to their unique sympathetic inhibitory effect.^12^

The research of the clinical applications of dexmedetomidine mainly focuses on surgical anesthesia, postoperative sedation and analgesia, while its pharmacological effects are mainly manifested in the protection of nervous system, immune system, body inflammation and organ function. There are many factors in the perioperative period that may lead to myocardial injury.^10^ The application research of dexmedetomidine in the perioperative period mainly focuses on the effect of the drug on hemodynamics and the protective effect on the liver, kidney, and brain. At the same time, the research on the protection of the heart is scarce.^3^ Myocardial injury is related to decreased blood return to the heart, decreased myocardial oxygen supply, inflammatory factors, endotoxins etc., but the exact mechanism is still unclear.^13^

Dexmedetomidine has a good protective effect on ischemia-reperfusion injury of various organs and tissues that is mainly related to the inhibition of lipid peroxidation mediated by oxygen free radicals. The study of Erer D et al^14^ showed that dexmedetomidine could reduce liver injury in rats with myocardial ischemia-reperfusion injury. Dong X et al^15^ confirmed the difference in the expression of oxidative stress indicators such as superoxide dismutase, catalase, glutathione, malon-dialdehyde, and oxidized protein in muscle specimens of ischemia-reperfusion injury model rats that received 25 μg/kg dexmedetomidine. Our results show that the proportion of patients with intraoperative systolic blood pressure greater than 180 and heart rate higher than 110 in the HTG was higher than that in the low troponin group. These results indicate that heart rate and blood pressure may be the key factors affecting the cardioprotection effect of dexmedetomidine. We may speculate that heart rate and blood pressure impact the protective effect of dexmedetomidine on ischemia-reperfusion injury by affecting hemodynamics.

The excessive release of inflammatory factors is closely related to myocardial injury. Dexmedetomidine reduces oxidative stress and inflammatory response in surgical patients, improves immune function and reduces surgical complications. Intraoperative inflammation can also affect the protective effect of dexmedetomidine on the heart.^16^ Studies have found that the use of dexmedetomidine in the perioperative period can effectively inhibit the level of oxidative stress and inflammatory response in patients, improve immune function and reduce postoperative complications.^17^

A meta-analysis by Wang et al^1^ included 67 clinical studies with a total of 4842 patients and found decreased epinephrine, norepinephrine, and cortisol release and decreased release of epinephrine, norepinephrine, and cortisol in patients receiving perioperative dexmedetomidine infusion. Levels of C-reactive protein, cytokines such as interleukin (IL)-6, tumor necrosis factor-α, etc. decreased, while the number of natural killer cells, B cells and CD4+ T cells and the ratio of CD4+:CD8+ and Th1:Th2 significantly increased, suggesting a decrease in the level of oxidative stress and inflammation and an enhancement of immune function in patients.^18^ A prospective randomized controlled study has also confirmed that the use of dexmedetomidine in patients undergoing coronary artery bypass grafting lowered the levels of inflammatory factors IL-1, IL-6, etc., and had an overall anti-inflammatory effect.^19^

The results of our study also showed that the neutrophil/lymphocyte ratio of the patients with elevated troponin was higher than that of the patients with normal troponin. It shows that intraoperative inflammation can also affect the cardioprotective effect of dexmedetomidine. In addition, we observed increased postoperative pain scores and length of hospital stay in the HTG of patients. Our results indicate that increased blood pressure, increased heart rate and increased neutrophil/lymphocyte ratio during the operation may also affect the analgesic effect of dexmedetomidine.

### Limitations:

It is a retrospective study with a high variability in the number of patients in each group. We only analyzed one indicator, troponin, which may impact the validity of our conclusions on the cardioprotection proprieties of dexmedetomidine. In addition, there are many interference factors that were not evaluated in this study, such as patients were on remifentanil as well, which decreased blood pressure and heart rate with analgesic properties as well. The use of remifentanil can result in cardi-protection and decrease in troponin levels, but may potentially have an impact on the results.

## CONCLUSION

Intraoperative systolic blood pressure, maximum heart rate, and postoperative neutrophil/lymph ratio are important factors that affect the myocardial protection proprieties of dexmedetomidine and may affect the postoperative analgesia effect and the length of hospital stay.

### Authors’ contributions:

**HL:** Conceived and designed the study.

**HL, CL and HS:** Collected the data and performed the analysis.

**HL, JL and HS:** Was involved in the writing of the manuscript and is responsible for the integrity of the study.

All authors have read and approved the final manuscript.
